# Association between serum selenium level and conversion of bacteriological tests during antituberculosis treatment* **

**DOI:** 10.1590/S1806-37132014000300010

**Published:** 2014

**Authors:** Milena Lima de Moraes, Daniela Maria de Paula Ramalho, Karina Neves Delogo, Pryscila Fernandes Campino Miranda, Eliene Denites Duarte Mesquita, Hedi Marinho de Melo Guedes de Oliveira, Antônio Ruffino-Netto, Paulo César de Almeida, Rachel Ann Hauser-Davis, Reinaldo Calixto Campos, Afrânio Lineu Kritski, Martha Maria de Oliveira

**Affiliations:** McGill University, Montreal, QC, Canada; Graduate Program in Clinical Medicine, Federal University of Rio de Janeiro School of Medicine, Rio de Janeiro, Brazil; Graduate Program in Clinical Medicine, Federal University of Rio de Janeiro School of Medicine, Rio de Janeiro, Brazil; Graduate Program in Clinical Medicine, Federal University of Rio de Janeiro School of Medicine, Rio de Janeiro, Brazil; Tuberculosis Research Center, Academic Program in Tuberculosis, Federal University of Rio de Janeiro School of Medicine, Rio de Janeiro, Brazil; Tuberculosis Research Center, Academic Program in Tuberculosis, Federal University of Rio de Janeiro School of Medicine, Rio de Janeiro, Brazil; University of São Paulo at Ribeirão Preto School of Medicine, Ribeirão Preto, Brazil; Graduate Course in Nutrition, Ceará State University, Fortaleza, Brazil; Pontifical Catholic University of Rio de Janeiro, Rio de Janeiro, Brazil; Department of Chemistry, Pontifical Catholic University of Rio de Janeiro, Rio de Janeiro, Brazil; Academic Program in Tuberculosis, Federal University of Rio de Janeiro School of Medicine, Rio de Janeiro, Brazil; Tuberculosis Research Center, Academic Program in Tuberculosis, Federal University of Rio de Janeiro School of Medicine, Rio de Janeiro, Brazil

**Keywords:** Selenium, Nutritional status, Tuberculosis, Immunity

## Abstract

**Objective:**

To determine whether serum selenium levels are associated with the conversion of bacteriological tests in patients diagnosed with active pulmonary tuberculosis after eight weeks of standard treatment.

**Methods:**

We evaluated 35 healthy male controls and 35 male patients with pulmonary tuberculosis, the latter being evaluated at baseline, as well as at 30 and 60 days of antituberculosis treatment. For all participants, we measured anthropometric indices, as well as determining serum levels of albumin, C-reactive protein (CRP) and selenium. Because there are no reference values for the Brazilian population, we used the median of the serum selenium level of the controls as the cut-off point. At 30 and 60 days of antituberculosis treatment, we repeated the biochemical tests, as well as collecting sputum for smear microscopy and culture from the patients.

**Results:**

The mean age of the patients was 38.4 ± 11.4 years. Of the 35 patients, 25 (71%) described themselves as alcoholic; 20 (57.0%) were smokers; and 21 (60.0%) and 32 (91.4%) presented with muscle mass depletion as determined by measuring the triceps skinfold thickness and arm muscle area, respectively. Of 24 patients, 12 (39.2%) were classified as moderately or severely emaciated, and 15 (62.5%) had lost > 10% of their body weight by six months before diagnosis. At baseline, the tuberculosis group had lower serum selenium levels than did the control group. The conversion of bacteriological tests was associated with the CRP/albumin ratio and serum selenium levels 60 days after treatment initiation.

**Conclusions:**

Higher serum selenium levels after 60 days of treatment were associated with the conversion of bacteriological tests in pulmonary tuberculosis patients.

## Introduction

The World Health Organization considers tuberculosis a serious public health problem. In 2010, 9.4 million new tuberculosis cases occurred, with 1.7 million associated deaths, among which 500,000 were HIV-positive patients. In Brazil, tuberculosis is the leading cause of mortality among patients with HIV/AIDS, a result arising from late diagnosis.^(^ ^1^ ^)^ Since 2006, the Global Plan to Stop TB has been prioritizing the critical points in the field of tuberculosis, especially the development of new diagnostic tests, vaccines, drugs, and biomarkers of therapeutic response, of healing, and of disease recurrence.^(^ ^2^ ^)^

Among the risk factors associated with the occurrence of tuberculosis are precarious working conditions and changes in host defense against the infection by *Mycobacterium tuberculosis*, such as malnutrition, smoking, diabetes mellitus, and alcohol abuse.^(^ ^3^ ^)^

The degree of malnutrition is associated with the severity of pulmonary tuberculosis in adults. Tuberculosis patients usually present malnutrition and a decrease in micronutrient levels, regardless of their HIV status.^(^ ^4^ ^)^

Recently, one group of authors^(^ ^5^ ^)^ reported that a two-month intervention with vitamin E and selenium supplements reduced oxidative stress and increased total antioxidant capacity in patients with pulmonary tuberculosis undergoing standard treatment. A similar improvement in the immune status of patients with tuberculosis who received selenium supplementation was also reported in another study.^(^ ^6^ ^)^

The objective of the present study was to determine whether serum selenium levels are associated with the conversion of bacteriological tests in patients diagnosed with active pulmonary tuberculosis after eight weeks of standard treatment. The conversion (to negative) of cultures of sputum collected eight weeks after treatment initiation has been used as a useful marker of the sterilizing activity of tuberculosis treatment,^(^ ^7^ ^)^ and a substantial improvement in serum selenium levels in these patients would indicate that selenium can be a biomarker of therapeutic response.

## Methods

**Study subjects**

Between March of 2007 and March of 2008, we included male patients with pulmonary tuberculosis admitted to either of the two referral hospitals for tuberculosis in the city of Rio de Janeiro, Brazil, namely the *Hospital Estadual Santa Maria* and the *Instituto Estadual de Doenças do Tórax Ary Parreiras*. We decided to include only male patients in the study because the great majority of the patients treated in these hospitals are males, and the inclusion of very few female patients could become a confounding factor in the data analysis. The patients enrolled in the present study had been hospitalized for clinical reasons; however, in most cases, the duration of hospital stay was prolonged for at least 60 days due to social reasons. The inclusion criteria were as follows: being 19-60 years of age; having a positive culture for *tuberculosis* or positive smear microscopy in spontaneous sputum in association with chest X-rays and symptoms indicative of tuberculosis; receiving treatment with first-line antituberculosis drugs; not having diabetes mellitus or renal disease (undergoing peritoneal dialysis or hemodialysis); having tested negative for HIV; and reporting no comorbidities. 

Because there are no established reference values for selenium levels in serum for the Brazilian population, we determined the serum selenium levels of 35 HIV-negative healthy subjects residing in the city of Rio de Janeiro (using similar inclusion criteria) in order to define a cut-off point. All subjects gave written informed consent. The study was approved by the Research Ethics Committee of the Federal University of Rio de Janeiro (Protocol no. 004/05, of April 28, 2005). The patients enrolled in the pilot study were not included in the present study.

### Data collection

A pilot study was conducted in order to determine the adequacy of the questionnaire applied to the study subjects. The interviewers were trained regarding data collection. Anthropometric measurements taken by different interviewers showed a high level of inter-rater agreement (> 95%).

The pulmonary tuberculosis patients completed a questionnaire regarding demographic data, socioeconomic data, and tobacco use, as well as the criteria used in the Cut down, Annoyed, Guilty, and Eye-opener (CAGE) questionnaire.^(^ ^8^ ^)^ Anthropometric measurements were collected at baseline, as well as at 30 and 60 days after antituberculosis treatment initiation. Blood and sputum samples were also collected at the same time points. At 30- and 60-day sample collection time points, some of the patients no longer presented sputum production, and therefore no sputum smear microscopy/culture were performed for those patients. The healthy subjects also completed the questionnaire, underwent anthropometric assessment, and had their blood samples collected.

The anthropometric evaluation consisted of two body weight measurements using a calibrated platform scale with a stadiometer (Filizola, São Paulo, Brazil) with a sensitivity of 100 g and maximum weight of 150 kg. The subjects were weighed barefoot and wearing light clothing. Height was measured twice (stadiometer with a sensitivity of 0.5 cm and maximum height of 191 cm). 

The body mass index (BMI) was calculated by the formula weight/height2 and classified according to the World Health Organization recommendations: underweight, < 18.5 kg/m^2^; normal weight, 18.5-24.9 kg/m^2^; and overweight, ≥ 25.0 kg/m^2^.^(^ ^9^ ^)^ All measurements were collected in accordance with the techniques recommended by Gibson^(^ ^10^ ^)^ in order to avoid possible bias. The patients with pulmonary tuberculosis also reported their usual weight (in the last 6 months) so that their weight loss until the beginning of the study (baseline) could be estimated.

The triceps skinfold thickness (TST) was measured three times with an adipometer (Lange Beta Technology Inc., Cambridge, MD, USA) with a sensitivity of 0.5 mm. Measurements were taken at the midpoint of the back of the non-dominant arm, between the acromion and olecranon, with the subjects standing with their arms relaxed and extended alongside the body.

The measurement of arm circumference (AC) was performed twice, with a flexible and inelastic millimeter tape at the same height as the midpoint used for the TST measurement. After that, the arm muscle area (AMA) was calculated using the following equation^(^ ^11^ ^)^:

AMA (cm²) = [(AC(cm) − π × TST(mm) ÷ 10)² − 10]/4π.

Mean TST and AMA results were calculated, and the cut-off values used were those by Frisancho.^(^ ^12^ ^)^

Peripheral blood samples were collected in the morning with subjects fasting for 12 h. The samples were collected in metal- and EDTA-free tubes. The samples were centrifuged at 3,000 g for 15 min for further quantification of albumin, C-reactive protein (CRP), and selenium. All quantifications were performed immediately after sample collection, except for the determination of selenium levels. In this case, a portion of the serum obtained was stored at −70°C for later quantification.

Albumin quantification was determined colorimetrically (Advia^(r)^; Siemens Healthcare Diagnostics, Eschborn, Germany). According to the manufacturer, normal albumin values should range from 3.4 to 4.8 g/dL. CRP was measured by nephelometry using a CardioPhase hsCRP assay (Dade Behring Holding GmbH, Liederbach, Germany) and a BNII nephelometer (Siemens Healthcare, Indianapolis, IN, USA). According to the manufacturer, normal values lay below 0.3 mg/dL.

In the present study, we evaluated the CRP/albumin ratio as a substitute for the prognostic inflammatory nutritional index because it maintains the same diagnostic sensitivity regarding the levels of complication risks.^(^ ^13^ ^)^ According to one study, the levels of complication risks are as follows: no risk, if the ratio is < 0.4; low risk, from 0.4 to 1.1; medium risk, from 1.2 to 2.0; and high risk, > 2.0.^(^ ^13^ ^)^

The determination of selenium levels was performed by graphite furnace atomic absorption spectrometry, using a ZEEnit 60 spectrometer (Analytik Jena, Jena, Germany) equipped with a selenium hollow cathode lamp operating at a wavelength of 196.0 nm. After the thawing and homogenizing of the serum samples, 200 mL aliquots were transferred to polyethylene tubes, free of trace elements, and 1 mL of a 0.1% v/v Triton ×100 solution was added. This solution (10 mL) was used for the instrumental analysis, together with a mixture (10 mL) containing palladium (0.15% m/v) and magnesium (0.10% m/v) as matrix modifier. External calibration was performed with calibration solutions prepared in the stock solution, and the temperature protocol is shown in Table 1. All measurements were conducted at least in triplicate.

**Table 1:** - Temperature program used in order to determine selenium levels in serum.

Step	Temperature, °C	Ramp, °C/s	Duration, s
Drying	90	10	10
Drying	120	15	20
Pyrolysis	500	10	20
Pyrolysis	1,100	30	30
Auto zero	1,100	0	6
Atomization^a^	2,200	2,000	3
Cleaning	2,300	1,000	3

a Measurement.

Sputum samples of the subjects included in the study were collected in disposable vials. Smear microscopy and cultures for mycobacteria were performed in accordance with the recommendations by the Brazilian National Ministry of Health.^(^ ^14^ ^)^

Cultures contaminated by other microorganisms were designated as contaminated and considered negative in the data analysis. The strains were identified as *tuberculosis* on the basis of the characteristics of the colonies (rough, opaque, and creamy) and biochemical testing (ability to produce niacin, nitrate reduction, and thermal inactivation of catalase).^(^ ^14^ ^)^ In the present study, the individuals were diagnosed with pulmonary tuberculosis at baseline when cultures were positive for *tuberculosis* or when there were positive results in sputum smear microscopy associated with X-ray findings and symptoms indicative of tuberculosis. Patients who presented with X-ray findings and symptoms indicative of tuberculosis but negative cultures or smear results at baseline were not included in the study. 

Susceptibility testing was performed on the clinical specimens from 28 patients who had positive cultures using the method of proportions, which is considered the gold standard. In addition, we used the indirect proportion method (one strain per patient) in order to determine the susceptibility of the *tuberculosis* strains to isoniazid, rifampin, streptomycin, and ethambutol. All of the tested strains were susceptible to the drugs tested.

New sputum samples were collected 30 and 60 days after treatment initiation, and new smear microscopy testing and cultures for mycobacteria were performed. Depending on the results of the tests, the patients could be reallocated to either of the two groups: tuberculosis-positive (TB+) group, when smears or cultures were positive for *tuberculosis*; and tuberculosis-negative (TB−) group, when smears and cultures were negative for *tuberculosis*. The individuals who were unable to produce sputum spontaneously at the moments of collection were not included in either group.

### Statistical analysis

The Kolmogorov-Smirnov test was used in order to verify the normality of the variables, and the Levene test was used in order to determine the equality of variances. A logarithmic transformation was used for the variables that showed non-normal distribution. We used Tukey's test and Games-Howell test to compare pairs of groups with equal and different variances, respectively. When appropriate, ANOVA and Student's t-test were used in order to estimate differences between quantitative variables. To evaluate the association between categorical variables, we used the chi-square test with continuity correction when indicated. A p-value < 0.05 was considered significant. The Statistical Package for the Social Sciences, version 16.0 (SPSS Inc., Chicago, IL, USA), was used for data analysis.

## Results

**Figure 1:**
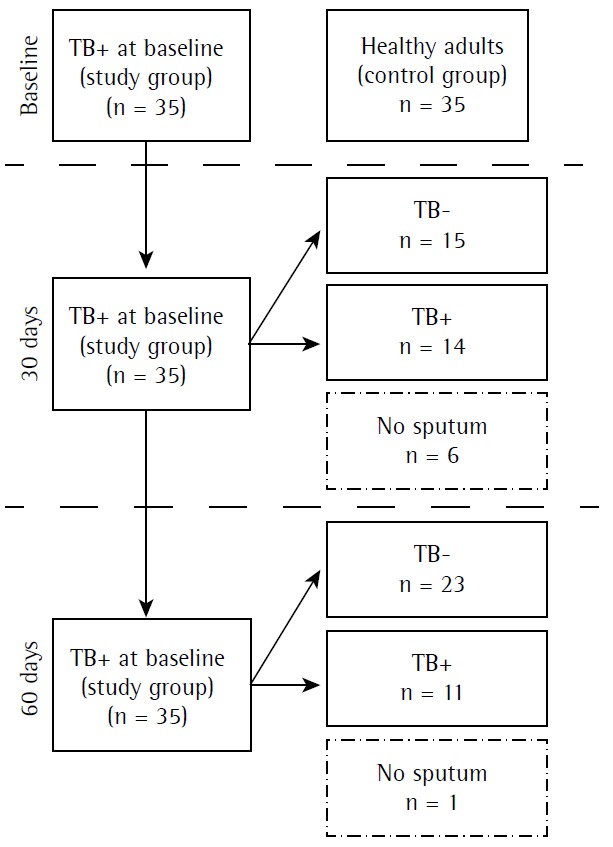
Study and control groups at baseline, at 30 days after antituberculosis treatment initiation, and at 60 days after antituberculosis treatment initiation. TB+: positive sputum culture or positive sputum smear microscopy results at that study time point; and TB−: negative sputum culture and negative sputum smear microscopy results at that study time point.

We included 35 pulmonary tuberculosis patients in the study group at baseline. Among these, 6 were recurrent tuberculosis patients. After 30 days of treatment, only 29 patients presented spontaneous sputum production, and, after 60 days of treatment, 34 patients showed spontaneous sputum production (Figure 1).

The general characteristics of the pulmonary tuberculosis patients are presented in Table 2. The mean age of the patients was 38.4 ± 11.4 years. Among the 35 male study subjects included in the study, 25 (71%) reported alcoholism according to the CAGE questionnaire, and 20 (57%) were smokers. We determined the BMI of 24 of the patients, and 12 (39%) were classified as being severely or moderately emaciated. Of the 35 patients, 21 (60%) and 32 (91%) were found to have with muscle mass depletion on the basis of their TST and AMA, respectively. Of the 24 patients who provided information regarding their weight by 6 months prior to their inclusion in the study, 15 (63%) had lost > 10% of their body weight. Statistically significant differences were found between the pulmonary tuberculosis patients and the healthy controls at baseline.

**Table 2:** - General characteristics of the patients with pulmonary tuberculosis (N = 35).a

Characteristic	Result
Age, years^b^	38.43 ± 11.42
Alcoholism	25 (71)
Smoking status	
Smokers	20 (57)
Former smokers	7 (20)
Never smokers	8 (23)
Weight loss, kg^b,c^	11.03 ± 9.69
Weight loss, %^c^	
> 10	15 (63)
5-10	4 (17)
< 5%	2 (8)
No loss	3 (12)
Classification according to BMI^d^	
Severe thinness	6 (19.4)
Moderate thinness	6(19.4)
Mild thinness	6 (19.4)
Normal weight	12 (38.7)
Overweight or obese	1 (3.2)
Classification according to TST	
Depletion	21 (60.0)
Normal	14 (40.0)
Classification according to AMA	
Depletion	32 (91.4)
Normal	3 (8.6)

BMI : body mass index

TST : triceps skinfold thickness

AMA : arm muscle area

a Values expressed as n (%), except where otherwise indicated.

b Values expressed as mean ± SD.

c n = 24.

d n = 3.

When we compared the three study time points (baseline, 30 days, and 60 days), we found that the conversion to a negative-culture status was associated with the CRP levels and the CRP/albumin ratio results at 30 and 60 days, as well as with albumin and selenium levels at 60 days (Table 3). No differences were observed between the TB+ and TB− groups for any of these variables at 30 days.

**Table 3:** - Anthropometric variables, biochemical test results, and serum selenium levels in the groups studied at the three study time points.a

Variable	Baseline		30 days after treatment initiation			60 days after treatment initiation		
	Control	TB+ group	TB− group	TB+ group	TB+ group at baseline	TB− group	TB+ group	TB+ group at baseline
	(n = 35)	(n = 35)	(n = 15)	(n = 14)	(n = 35)	(n = 23)	(n = 11)	(n = 35)
BMI, kg/m^2^	25.27 ± 3.59	18.21 ± 2.53*	19.60 ± 2.18*	19.40 ± 2.46*	19.49 ± 2.86*	20.64 ± 3.25*	20.41 ± 3.10*	20.53 ± 3.11*
TST, mm	12.71 ± 4.99	5.11 ± 2.51*	6.28 ± 2.48*	5.87 ± 1.82*	6.13 ± 2.57*	7.42 ± 4.32*	7.15 ± 2.83*	7.35 ± 3.80*
AMA, cm^2^	55.15 ± 12.11	26.10 ± 7.92*	28.54 ± 9.86*	29.07 ± 9.24*	28.74 ± 9.69*	32.29 ± 12.37*	30.52 ± 12.09*	31.77 ± 11.94*
Alb, g/dL	4.86 ± 0.19	3.64 ± 0.62*	3.99 ± 0.38*	4.02 ± 0.60*	4.02 ± 0.47*	4.27 ± 0.50*†	3.95 ± 0.37*	4.16 ± 0.48*†
CRP, mg/dL	0.16 ± 0.16	6.35 ± 4.12*	2.31 ± 1.88*†	4.33 ± 3.36*	3.66 ± 3.55*	1.95 ± 1.70*†	4.43 ± 3.69*‡	2.68 ± 2.71*†
CRP/alb ratio	0.03 ± 0.03	1.93 ± 1.58*	0.60 ± 0.52*†	1.22 ± 1.20*	0.99 ± 1.11*†	0.48 ± 0.44*†	1.20 ± 1.06*‡	0.70 ± 0.76
Se, μg/L	100.12 ± 12.11	80.13 ± 46.92*	93.55 ± 56.40*	77.31 ± 40.64*	88.26 ± 54.56*	104.53 ± 55.35	70.89 ± 38.66*‡	97.60 ± 54.59††

TB+ : positive sputum culture or positive sputum smear microscopy results at that study time point

TB− : negative sputum culture and negative smear sputum microscopy results at that study time point

BMI : body mass index

TST : triceps skinfold thickness

AMA : arm muscle area

Alb : albumin

CRP : C-reactive protein

Se : selenium

a Values expressed as mean ± SD. *p < 0.05 vs. control. †p < 0.05 vs. TB+ group at baseline. ‡p < 0.05 TB+ group vs. TB− group. ††p < 0.05 TB+ group vs. TB+ group at baseline. Tukey test (equal variances), Games-Howell test (different variances).

Table 4 presents the distribution of patients in the TB+ and TB− groups in relation to the results of the biochemical tests and serum selenium levels at the three study time points in order to determine the existence of any associations. In order to evaluate the association between the results of bacteriological tests (culture and smear microscopy) and serum selenium levels, we used the cut-off point based on the median of the results obtained in the healthy control group. 

**Table 4:** - Distribution of the patients in the TB+ and TB− groups in relation to the results of the biochemical tests and serum selenium levels at the three study time points.a

Variable	Baseline	30 days after treatment initiation			60 days after treatment initiation		
	TB+ group	TB− group	TB+ group	p*	TB− group	TB+ group	p*
	(n = 35)	(n = 15)	(n = 14)		(n = 23)	(n = 11)	
Albumin, g/dL				0.792			0.338
< 3.4	11 (100)	1 (33.3)	2 (66.7)		2 (100)	0 (0.0)	
3.4-4.8b	24 (100)	13 (54.2)	11 (45.8)		19 (63.3)	11 (36.7)	
>4.8	0 (0.0)	1 (50.0)	1 (50.0)		2 (100)	0 (0.0)	
CRP, mg/dL				0.617			0.683
< 0.3b	1 (100)	2 (100)	0 (0.0)		2 (100)	0 (0.0)	
≥ 0.3	34 (100)	13 (50.0)	13 (50.0)		21 (65.6)	11 (34.4)	
CRP/albumin ratioc				0.206			0.041
< 0.4	2 (100)	3 (30.0)	7 (70.0)		12 (75.0)	4 (25.0)	
0.4-1.1	11 (100)	5 (50.0)	5 (50.0)		9 (81.8)	2 (18.2)	
1.2-2.0	8 (100)	2 (50.0)	2 (50.0)		2 (50.0)	2 (50.0)	
> 2.0	13 (100)	3 (100)	0 (0.0)		0 (0.0)	3 (100)	
Selenium							
< cut-off pointd	24 (100)	9 (47.4)	10 (52.6)	0.518	12 (54.5)	10 (45.5)	0.027
≥ cut-off pointd	11 (100)	6 (60.0)	4 (40.0)		11 (91.7)	1 (8.3)	

TB− : negative sputum culture and negative sputum smear microscopy results at that study time point

TB+ : positive sputum culture or positive sputum smear microscopy results at that study time point

CRP : C-reactive protein

a Values expressed as n (%).

b Normal values.

c Used in order to determine the level of complication risks.(13)

d Based on the median of the results obtained in the healthy control group. *Chi-square test.

## Discussion

In the present study, the clinical characteristics of the patients are similar to those described in other studies carried out in referral hospitals for the treatment of tuberculosis in developing nations, with high rates of alcoholism and tobacco use.^(^ ^15^ ^)^

The relationship between tuberculosis and malnutrition has been revisited, since malnutrition may predispose to the development of active tuberculosis, and tuberculosis can contribute to malnutrition.^(^ ^16^ ^)^ The mean weight loss in the study group prior to antituberculosis treatment initiation was 11.03 ± 9.69 kg. This can be considered even more significant when categorized by the percentage of body weight loss, because 63% of the patients presented with a weight loss ≥ 10%, which is considered a predisposing factor for tuberculosis.^(^ ^17^ ^)^

In the present study, the assessment of the nutritional status based on anthropometric parameters (BMI, TST, and AMA) confirmed the depleted nutritional status in the study group, as described in the literature.^(^ ^18^ ^)^ For any infection, there is a complex interplay between the host response and the virulence of the microorganism, which modulates the metabolic response, as well as the degree and pattern of tissue loss. In tuberculosis patients, reduced appetite, malabsorption of macronutrients and micronutrients, and altered metabolism lead to cachexia.^(^ ^16^ ^)^ However, no association between the nutritional parameters studied and culture conversion at 60 days of antituberculosis treatment was observed. Nevertheless, we found that low BMI, TST, and AMA persisted in the tuberculosis patients (even in those whose results converted to negative) after 60 days of treatment.

The use of BMI as an indicator of nutrition in the relationship between nutritional status and tuberculosis has been reported.^(^ ^19^ ^)^ The evaluation of TST and AMA in patients with tuberculosis, however, is less often described in the literature. Nevertheless, one group of authors^(^ ^20^ ^)^ described differences in lean body mass and fat mass gain in tuberculosis patients after 6 months of treatment. This fact points to the importance of not only evaluating the overall weight gain, but also differentiating it between lean and fat body mass.

Regarding the biochemical tests studied, we found that albumin levels improved during antituberculosis treatment. Patients with newly diagnosed tuberculosis have been described to present with lower albumin levels when compared with healthy control groups,^(^ ^18^ ^)^ which corroborates the results in the present study. In a study in Tanzania, the albumin levels of patients with tuberculosis also increased significantly after 60 days of antituberculosis treatment, equaling to the levels found in the control group, which is at odds with our findings.^(^ ^21^ ^)^ In another study conducted in Brazil, tuberculosis patients were followed for 6 months, and no improvement in albumin levels throughout the study was observed.^(^ ^22^ ^)^

Higher levels of albumin have been considered as a predictor of a better outcome in patients with pulmonary tuberculosis. Albumin has also been identified as an indicator of protein status when tuberculosis is diagnosed.^(^ ^23^ ^)^ However, cytokines present during the acute phase response (APR) to the infection can suppress the synthesis of albumin, thereby reducing its circulating levels. Therefore, it is difficult to interpret low albumin levels in patients with active tuberculosis without other parameters to assess APR and malnutrition, since low albumin levels may reflect both APR to infection and protein deficiency. Thus, the discrepancy across studies might be due to variations in nutritional status, the intensity of APR in the studied populations, or the small number of patients included.

Because CRP synthesis is increased in the host systemic response to infection, statistically significant differences were observed between the TB+ and TB− groups at baseline, at 30 days of treatment, and at 60 days of treatment, confirming the association between bacteriological conversion and decreased in CRP levels.

One group of authors evaluated CRP levels in patients with pulmonary tuberculosis during 6 months of treatment; at 3 and 6 months after treatment initiation, there was a significant reduction in CRP levels.^(^ ^22^ ^)^ CRP has been identified as an important indicator in the diagnosis of individuals with suspected tuberculosis and positive smear microscopy.^(^ ^24^ ^)^ In our study, a statistically significant association between lower CRP/albumin ratio values and negative cultures for mycobacteria was also found. The CRP/albumin ratio has been described to be increased in patients with other APR-related diseases.^(^ ^14^ ^)^

The tuberculosis infection is a condition known to induce oxidative stress in the infected organism, such as the production of reactive oxygen species (ROS) derived from free radicals. These ROS are associated with dysfunction in pulmonary tuberculosis. A way of suppressing these ROS is by means of antioxidant enzymes, which scavenge free radicals and protect cells from oxidative damage. Various of these enzymes, such as glutathione peroxidase, have selenium as an essential element.^(^ ^25^ ^)^ Thus, a reduction in micronutrient intake (such as vitamins, zinc, and selenium) leads to impaired immune responses.

Studies show that patients with active tuberculosis have lower concentrations of various micronutrients, including selenium, in blood.^(^ ^26^ ^)^ In the present study, the healthy subjects showed higher selenium levels when compared with the study group at baseline. Among the pulmonary tuberculosis patients, we found an association between positive culture results and low selenium levels even after 60 days of treatment. Micronutrient deficiency is a frequent cause of secondary immunodeficiency and morbidity due to related infections, including tuberculosis. This trace element has an important role in the maintenance of immune processes and, therefore, may have a fundamental role in the defense against the mycobacteria. Low selenium levels have been considered a significant risk factor for the development of mycobacterial disease in HIV-positive patients.^(^ ^27^ ^)^ In one study with 22 pulmonary tuberculosis patients who were newly diagnosed with positive sputum,^(^ ^28^ ^)^ the authors found a significant difference between selenium levels between the control and study groups at baseline, as we found in the present study. However, in that study, no bacteriological tests were performed 60 days later. In the present study, it is noteworthy that the selenium levels remained low in the TB+ group individuals. One group of authors in India evaluated the circulating concentrations of antioxidant enzymes that have selenium as an essential component and are markers of oxidative stress in patients with pulmonary tuberculosis.^(^ ^29^ ^)^ The results showed lower antioxidant potential as determined by low levels of superoxide dismutase, catalase, and glutathione, as well as increased lipid peroxidation (malonaldehyde), in the patients with tuberculosis. However, the antioxidant potential and selenoenzymes levels increased with the treatment, as observed in the present study.

In another study, conducted in Malawi^(^ ^30^ ^)^ and involving 500 newly diagnosed pulmonary tuberculosis patients (including 370 coinfected with HIV), it was observed that micronutrient deficiencies were common in all patients, and 88% of the sample was deficient in selenium. These decreased selenium concentrations were also associated with the severity of anemia, which is common in active tuberculosis patients. It is thus suggested that selenium deficiency might contribute to anemia via increased oxidative stress in tuberculosis patients. According to one group of authors,^(^ ^5^ ^)^ a two-month intervention with vitamin E and selenium supplementation reduced oxidative stress and increased the total antioxidant capacity in patients with treated pulmonary tuberculosis. However, in that study,^(^ ^5^ ^)^ the association between selenium supplementation and negative smear microscopy results or cultures at the end of 2 months of treatment was not reported.

In summary, in our study, we found poor nutritional status (based on BMI, TST, and AMA) in patients with pulmonary tuberculosis, but these parameters were not associated with sputum culture conversion at 60 days of antituberculosis treatment. The relationship between CRP and albumin levels might be a useful tool for assessing the bacteriological conversion in patients with tuberculosis. In addition, low serum selenium levels after 60 days of treatment were associated with positive sputum culture and positive sputum smear microscopy. Our results corroborate the findings in other studies that showed improvement of the immune status of tuberculosis patients who received selenium supplementation.^(^ ^27^ ^,^ ^30^ ^)^ Thus, despite the limitations of the present study (small sample of tuberculosis patients and inclusion of male patients only), our results suggest that selenium levels and CRP/albumin ratio can be used as biomarkers of therapeutic response in pulmonary tuberculosis. Further studies are necessary in order to confirm or refute our results. In addition, studies on the interaction between *tuberculosis* and serum selenium levels are needed in order to help us understand whether (and how) tuberculosis modulates selenium levels.
