# Supplementary material for: Association between serum selenium level and conversion of bacteriological tests during antituberculosis treatment
Source: J Bras Pneumol. 2014 May-Jun;40(3):269–78. doi: 10.1590/S1806-37132014000300010 (PMC4109199; doi:10.1590/S1806-37132014000300010)
Supplement: Translation

# Translation: Associações entre níveis de selênio sérico e conversão de testes bacteriológicos durante o tratamento antituberculose*

**Authors:** Milena Lima de Moraes, Daniela Maria de Paula Ramalho, Karina Neves Delogo, Pryscila Fernandes Campino Miranda, Eliene Denites Duarte Mesquita, Hedi Marinho de Melo Guedes de Oliveira, Antônio Ruffino-Netto, Paulo César de Almeida, Rachel Ann Hauser-Davis, Reinaldo Calixto Campos, Afrânio Lineu Kritski, Martha Maria de Oliveira

**Affiliations:** McGill University, Montreal, QC, Canadá; Programa de Pós-Graduação em Clínica Médica, Faculdade de Medicina da Universidade Federal do Rio de Janeiro, Rio de Janeiro (RJ) Brasil; Programa de Pós-Graduação em Clínica Médica, Faculdade de Medicina da Universidade Federal do Rio de Janeiro, Rio de Janeiro (RJ) Brasil; Programa de Pós-Graduação em Clínica Médica, Faculdade de Medicina da Universidade Federal do Rio de Janeiro, Rio de Janeiro (RJ) Brasil; Centro de Pesquisa em Tuberculose, Programa Acadêmico de Tuberculose, Faculdade de Medicina da Universidade Federal do Rio de Janeiro, Rio de Janeiro (RJ) Brasil; Centro de Pesquisa em Tuberculose, Programa Acadêmico de Tuberculose, Faculdade de Medicina da Universidade Federal do Rio de Janeiro, Rio de Janeiro (RJ) Brasil; Faculdade de Medicina de Ribeirão Preto, Universidade de São Paulo, Ribeirão Preto (SP) Brasil; Curso de Pós-Graduação em Nutrição, Universidade Estadual do Ceará, Fortaleza, Brasil; Pontifícia Universidade Católica do Rio de Janeiro, Rio de Janeiro (RJ) Brasil; Departamento de Química, Pontifícia Universidade Católica do Rio de Janeiro, Rio de Janeiro (RJ) Brasil; Programa Acadêmico de Tuberculose, Faculdade de Medicina da Universidade Federal do Rio de Janeiro, Rio de Janeiro (RJ) Brasil; Centro de Pesquisa em Tuberculose, Programa Acadêmico de Tuberculose, Faculdade de Medicina da Universidade Federal do Rio de Janeiro, Rio de Janeiro (RJ) Brasil

**Keywords:** Selênio, Estado nutricional, Tuberculose, Imunidade

## Abstract

**Objetivo:**

Determinar se os níveis séricos de selênio estão associados à conversão dos testes bacteriológicos em pacientes diagnosticados com tuberculose pulmonar ativa após oito semanas de tratamento-padrão.

**Métodos:**

No início do estudo, avaliamos 35 controles saudáveis, do sexo masculino, e 35 pacientes do sexo masculino com tuberculose pulmonar. Estes foram também avaliados após 30 e 60 dias de tratamento antituberculose. Todos os participantes submeteram-se a medições antropométricas e quantificação dos níveis séricos de albumina, proteína C reativa (PCR) e selênio. Como não há valores de referência para a população brasileira, usamos a mediana dos resultados de selênio sérico dos controles como ponto de corte. Aos 30 e 60 dias do tratamento antituberculose, todos os testes bioquímicos foram repetidos, e foram coletadas amostras de escarro para baciloscopia e cultura.

**Resultados:**

A média de idade dos pacientes foi de 38,4 ± 11.4 anos. Dos 35 pacientes, 25 (71,0%) referiram alcoolismo, 20 (57,0%) eram fumantes, e 21 (60,0%) e 32 (91,4%) apresentavam depleção muscular pela medição da dobra cutânea tricipital e da área muscular do braço, respectivamente. De 24 pacientes, 12 (39,2%) foram classificados em moderadamente ou gravemente magros, e 15 (62,5%) apresentaram perda de peso > 10% em até seis meses antes do diagnóstico. No início do estudo, o grupo com tuberculose apresentou menores níveis de selênio sérico que os controles. A conversão dos testes bacteriológicos associou-se à relação PCR/albumina e aos níveis de selênio sérico 60 dias após o início do tratamento.

**Conclusões:**

Níveis maiores de selênio sérico após 60 dias de tratamento associaram-se à conversão bacteriológica em pacientes com tuberculose pulmonar.

## Introdução

A Organização Mundial da Saúde considera a tuberculose um sério problema de saúde pública. Em 2010, houve 9,4 milhões de novos casos de tuberculose, com 1,7 milhões de mortes associadas, dentre os quais 500.000 eram pacientes HIV positivos. No Brasil, a tuberculose é a principal causa de mortalidade em pacientes com HIV/AIDS, em consequência do diagnóstico tardio.^(^ ^1^ ^)^ Desde 2006, o Global Plan to Stop TB vem priorizando os pontos críticos na área da tuberculose, especialmente o desenvolvimento de novos testes diagnósticos, vacinas, medicamentos e biomarcadores de resposta terapêutica, cura e recidiva.^(^ ^2^ ^)^

Dentre os fatores de risco de tuberculose estão precárias condições de trabalho e alterações na defesa do hospedeiro contra a infecção por *Mycobacterium tuberculosis,* tais como desnutrição, tabagismo, diabetes mellitus e abuso de álcool.^(^ ^3^ ^)^

O grau de desnutrição está relacionado com a gravidade da tuberculose pulmonar em adultos. Pacientes com tuberculose geralmente apresentam desnutrição e diminuição dos níveis de micronutrientes, independentemente de serem HIV positivos ou não.^(^ ^4^ ^)^

Recentemente, um grupo de autores^(^ ^5^ ^)^ relatou que uma intervenção de dois meses com vitamina E e suplementos de selênio reduziu o estresse oxidativo e aumentou a capacidade antioxidante total em pacientes com tuberculose pulmonar submetidos ao tratamento-padrão. Relatou-se em outro estudo melhoria semelhante no estado imunológico de pacientes com tuberculose que receberam suplemento de selênio.^(^ ^6^ ^)^

O objetivo do presente estudo foi determinar se os níveis séricos de selênio estão relacionados com a conversão de testes bacteriológicos em pacientes com diagnóstico de tuberculose pulmonar ativa após oito semanas de tratamento-padrão. A conversão (de resultado positivo em negativo) de culturas de escarro coletado oito semanas após o início do tratamento tem sido usada como um marcador útil da atividade esterilizante do tratamento da tuberculose,^(^ ^7^ ^)^ e uma melhora substancial nos níveis séricos de selênio nesses pacientes indicaria que o selênio pode ser um biomarcador de resposta terapêutica. 

## Métodos

### Participantes do estudo

Entre março de 2007 e março de 2008 foram incluídos no estudo pacientes do sexo masculino com tuberculose pulmonar internados em qualquer um dos dois hospitais de referência para o tratamento da tuberculose no Rio de Janeiro (RJ), o Hospital Estadual Santa Maria e o Instituto Estadual de Doenças do Tórax Ary Parreiras. Decidimos incluir apenas pacientes do sexo masculino porque a grande maioria dos pacientes tratados nos hospitais supracitados é do sexo masculino, e a inclusão de pouquíssimos pacientes do sexo feminino poderia se tornar um fator de confusão na análise dos dados. Os pacientes incluídos no presente estudo haviam sido hospitalizados por motivos clínicos; no entanto, na maioria dos casos, a internação hospitalar prolongou-se até 60 dias no mínimo, por motivos sociais. Os critérios de inclusão foram os seguintes: ter 19-60 anos de idade; apresentar cultura positiva para *M. tuberculosis* ou resultado positivo de baciloscopia de escarro espontâneo com radiografia de tórax e sintomas indicativos de tuberculose; receber tratamento com tuberculostáticos de primeira linha; não apresentar diabetes mellitus ou doença renal (diálise peritoneal ou hemodiálise); apresentar sorologia negativa para HIV e não relatar comorbidades. 

Como não existem valores de referência estabelecidos para os níveis séricos de selênio na população brasileira, quantificamos, por meio de critérios de inclusão semelhantes, os níveis séricos de selênio em 35 indivíduos saudáveis HIV negativos residentes na cidade do Rio de Janeiro, a fim de definir um ponto de corte. Todos os participantes assinaram um termo de consentimento livre e esclarecido. O estudo foi aprovado pelo Comitê de Ética em Pesquisa da Universidade Federal do Rio de Janeiro (Protocolo nº 004/05, de 28 de abril de 2005). Os pacientes incluídos no estudo piloto não foram incluídos no presente estudo. 

### Coleta de dados

Um estudo piloto foi realizado a fim de determinar a adequação do questionário aplicado aos participantes do presente estudo. Os entrevistadores foram treinados quanto à coleta de dados. Medidas antropométricas realizadas por diferentes entrevistadores apresentaram um alto nível de concordância interobservador (> 95%). 

Os pacientes com tuberculose pulmonar responderam a um questionário sobre dados demográficos, dados socioeconômicos e uso de tabaco, bem como os critérios usados no questionário *Cut down, Annoyed, Guilty, and Eye-opener* (CAGE, Reduzir, Aborrecido, Culpado e Bebeu de manhã).^(^ ^8^ ^)^ Foram realizadas medidas antropométricas no início do estudo, bem como 30 e 60 dias após o início do tratamento da tuberculose. Amostras de sangue e escarro foram também coletadas nos mesmos momentos. Nos momentos de coleta de amostras aos 30 e 60 dias, alguns dos pacientes não mais apresentavam produção de escarro e, portanto, não se realizou baciloscopia/cultura de escarro nesses pacientes. Os indivíduos saudáveis também responderam ao questionário, foram submetidos a avaliação antropométrica e forneceram amostras de sangue. 

A avaliação antropométrica consistiu em duas medidas de peso corporal em uma balança de plataforma calibrada com um estadiômetro (Filizola, São Paulo, Brasil), com sensibilidade de 100 g e peso máximo de 150 kg. Os indivíduos foram pesados descalços e com roupas leves. A estatura foi medida duas vezes (estadiômetro com sensibilidade de 0,5 cm e altura máxima de 191 cm). 

O índice de massa corporal (IMC) foi calculado pela fórmula peso/estatura2 e classificado de acordo com as recomendações da Organização Mundial da Saúde: baixo peso, < 18,5 kg/m^2^; peso normal, 18,5-24,9 kg/m^2^; sobrepeso, ≥ 25,0 kg/m^2^.(9) Todas as medidas foram realizadas de acordo com as técnicas recomendadas por Gibson^(^ ^10^ ^)^ para evitar um possível viés. Os pacientes com tuberculose pulmonar também relataram seu peso habitual (nos últimos 6 meses) de modo que sua perda de peso até ao início do estudo (perda de peso basal) pudesse ser estimada. 

A dobra cutânea tricipital (DCT) foi medida três vezes com um adipômetro (Lange Beta Technology Inc., Cambridge, MD, EUA) com sensibilidade de 0,5 mm. As medidas foram realizadas no ponto médio da parte de trás do braço não dominante, entre o acrômio e o olécrano, com os participantes em pé com os braços relaxados e estendidos ao longo do corpo. 

A circunferência do braço (CB) foi medida duas vezes, com uma fita milimétrica flexível e inelástica, na mesma altura que o ponto médio usado para medir a DCT. Posteriormente, a área muscular do braço (AMB) foi calculada por meio da seguinte equação^(^ ^11^ ^)^: 

AMB (cm²) = [(CB(cm) − π × DCT(mm) ÷ 10)² − 10]/4π.

Foram calculadas as médias de DCT e AMB, e os valores de corte usados foram os de Frisancho.^(^ ^12^ ^)^ Amostras de sangue periférico foram coletadas no período da manhã, com os participantes em jejum de 12 h. As amostras foram coletadas em tubos sem metal ou EDTA. As amostras foram centrifugadas a 3.000 g durante 15 min para posterior quantificação de albumina, proteína C reativa (PCR) e selênio. Todas as quantificações foram realizadas imediatamente após a coleta das amostras, exceto a quantificação dos níveis de selênio. Nesse caso, uma parte do soro obtido foi armazenada a −70°C para posterior quantificação. 

A quantificação da albumina foi realizada colorimetricamente (Advia^(r)^; Siemens Healthcare Diagnostics, Eschborn, Alemanha). De acordo com o fabricante, valores normais de albumina variam de 3,4 a 4,8 g/dL. A PCR foi medida por nefelometria, com o ensaio CardioPhase hsCRP (Dade Behring Holding GmbH, Liederbach, Alemanha) e um nefelômetro BNII (Siemens Healthcare, Indianápolis, IN, EUA). De acordo com o fabricante, valores abaixo de 0,3 mg/dL são normais. 

No presente estudo, a relação PCR/albumina foi avaliada como substituta do índice prognóstico inflamatório nutricional porque mantém a mesma sensibilidade diagnóstica no tocante aos riscos de complicações.^(^ ^13^ ^)^ De acordo com um estudo, os riscos de complicações são os seguintes: nenhum risco, se a relação for < 0,4; baixo risco, se a relação variar de 0,4 a 1,1; risco médio, se a relação variar de 1,2 a 2,0 e alto risco, se a relação for > 2,0.^(^ ^13^ ^)^

Os níveis de selênio foram quantificados por meio de espectrometria de absorção atômica em forno de grafite, com um espectrômetro ZEEnit 60 (Analytik Jena, Jena, Alemanha) equipado com uma lâmpada de cátodo oco de selênio com comprimento de onda de 196,0 nm. Após o descongelamento e a homogeneização das amostras de soro, alíquotas de 200 mL foram transferidas para tubos de polietileno, sem elementos traços, e foi adicionado 1 mL de uma solução de Triton ×100 a 0,1% v/v. Essa solução (10 mL) foi usada para a análise instrumental, com uma mistura (10 mL) de paládio (0,15% m/v) e magnésio (0,10% m/v) como modificadora de matriz. A calibragem externa foi realizada com soluções de calibragem preparadas na solução estoque, e o protocolo de temperatura é apresentado na Tabela 1. Todas as medições foram realizadas no mínimo três vezes.

**Tabela 1:** Temperaturas usadas para determinar os níveis séricos de selênio.

Passo	Temperatura, °C	Rampa, °C/s	Duração, s
Secagem	90	10	10
Secagem	120	15	20
Pirólise	500	10	20
Pirólise	1.100	30	30
Autozero	1.100	0	6
Atomização^a^	2.200	2.000	3
Limpeza	2.300	1.000	3

a Medida.

As amostras de escarro dos indivíduos incluídos no estudo foram coletadas em frascos descartáveis. A baciloscopia e as culturas para micobactérias foram realizadas de acordo com as recomendações do Ministério da Saúde.^(^ ^14^ ^)^

Culturas contaminadas por outros microrganismos foram denominadas contaminadas e consideradas negativas na análise dos dados. As cepas foram identificadas como *M. tuberculosis* com base nas características das colônias (ásperas, opacas e cremosas) e em testes bioquímicos (capacidade de produzir niacina, redução de nitrato e termoinativação da catalase).^(^ ^14^ ^)^ No presente estudo, os indivíduos receberam diagnóstico de tuberculose pulmonar no início do estudo quando as culturas foram positivas para *M. tuberculosis* ou quando houve resultados positivos de baciloscopia de escarro associados a achados radiológicos e sintomas indicativos de tuberculose. Não foram incluídos no estudo os pacientes que, embora apresentassem achados radiológicos e sintomas indicativos de tuberculose, apresentaram cultura ou baciloscopia negativa no início do estudo. 

O teste de sensibilidade foi realizado em espécimes clínicos provenientes de 28 pacientes com culturas positivas pelo método das proporções, que é considerado o padrão ouro. Além disso, usamos o método indireto das proporções (uma cepa por paciente) para determinar a sensibilidade das cepas de *M. tuberculosis* a isoniazida, rifampicina, estreptomicina e etambutol. Todas as cepas testadas mostraram-se sensíveis às drogas testadas. 

Novas amostras de escarro foram coletadas 30 e 60 dias após o início do tratamento, e foram realizados novos testes de baciloscopia e cultura para micobactérias. Dependendo dos resultados dos testes, os pacientes eram colocados em um dos seguintes grupos: o grupo positivo para tuberculose (TB+), quando os esfregaços ou as culturas eram positivos para *M. tuberculosis*, e o grupo negativo para tuberculose (TB−), quando os esfregaços e culturas eram negativos para *M. tuberculosis*. Os indivíduos que não foram capazes de produzir escarro espontaneamente nos momentos de coleta não foram incluídos em nenhum dos grupos. 

### Análise estatística

O teste de Kolmogorov-Smirnov foi usado para verificar a normalidade das variáveis, e o teste de Levene foi usado para verificar a igualdade das variâncias. A transformação logarítmica foi usada para as variáveis que não apresentaram distribuição normal. O teste de Tukey e o teste de Games-Howell foram usados para comparar pares de grupos com variâncias iguais e diferentes, respectivamente. Quando apropriado, a ANOVA e o teste t de Student foram usados para estimar diferenças entre variáveis quantitativas. Para avaliar a associação entre variáveis categóricas, foi usado o teste do qui-quadrado com correção de continuidade, quando necessário. Valores de p < 0,05 foram considerados significativos. Para a análise dos dados, foi usado o programa *Statistical Package for the Social Sciences,* versão 16.0 (SPSS Inc., Chicago, IL, EUA). 

## Resultados

Foram incluídos 35 pacientes com tuberculose pulmonar no grupo de estudo no início do estudo. Dos 35 pacientes, 6 eram pacientes com recidiva de tuberculose. Após 30 dias de tratamento, apenas 29 pacientes apresentaram produção espontânea de escarro, e, após 60 dias de tratamento, 34 pacientes apresentaram produção espontânea de escarro (Figura 1).

**Figura 1:** Grupo controle e grupo de estudo no início do estudo, 30 dias após o início do tratamento da tuberculose e 60 dias após o início do tratamento da tuberculose. TB+: cultura de escarro positiva ou baciloscopia de escarro positiva naquele momento do estudo; e TB−: cultura de escarro negativa e baciloscopia de escarro negativa naquele momento do estudo.

As características gerais dos pacientes com tuberculose pulmonar são apresentadas na Tabela 2. A média de idade dos pacientes foi de 38,4 ± 11,4 anos. Dos 35 pacientes, 25 (71%) relataram alcoolismo de acordo com o questionário CAGE, e 20 (57%) eram fumantes. Calculamos o IMC de 24 dos pacientes, e 12 (39%) foram classificados em moderadamente ou gravemente magros. A DCT revelou depleção de massa muscular em 21 (60%) dos 35 pacientes, e a AMB o fez em 32 (91%). Dos 24 pacientes que forneceram informações a respeito de seu peso até 6 meses antes de sua inclusão no estudo, 15 (63%) haviam perdido > 10% de seu peso corporal. Foram encontradas diferenças estatisticamente significantes entre os pacientes com tuberculose pulmonar e os controles saudáveis no início do estudo. 

**Tabela 2:** Características gerais dos pacientes com tuberculose pulmonar (N = 35).a

Característica	Resultado
Idade, anos^b^	38,43 ± 11,42
Alcoolismo	25 (71)
Tabagismo	
Fumantes	20 (57)
Ex-fumantes	7 (20)
Nunca fumaram	8 (23)
Perda de peso, kg^b,c^	11,03 ± 9,69
Perda de peso, %^c^	
> 10	15 (63)
5-10	4 (17)
< 5%	2 (8)
Sem perda	3 (12)
Classificação de acordo com o IMC^d^	
Magreza grave	6 (19,4)
Magreza moderada	6(19,4)
Magreza leve	6 (19,4)
Peso normal	12 (38,7)
Sobrepeso ou obesidade	1 (3,2)
Classificação de acordo com a DCT	
Depleção	21 (60,0)
Normal	14 (40,0)
Classificação de acordo com a AMB	
Depleção	32 (91,4)
Normal	3 (8,6)

IMC : índice de massa corporal

DCT : dobra cutânea tricipital

AMB : área muscular do braço

a Valores expressos em n (%), exceto onde indicado

b Valores expressos em média ± dp

c n = 24

d n = 31

Quando comparamos os três momentos do estudo (início do estudo, 30 dias e 60 dias), constatamos que a conversão da cultura em cultura negativa relacionou-se com os níveis de PCR e a relação PCR/albumina aos 30 e 60 dias, bem como com os níveis de albumina e selênio aos 60 dias (Tabela 3). Não foram observadas diferenças entre os grupos TB+ e TB− quanto a qualquer uma dessas variáveis aos 30 dias.

**Tabela 3:** Variáveis antropométricas, resultados de testes bioquímicos e níveis séricos de selênio nos grupos estudados nos três momentos do estudo.a

Variável	Início do estudo		30 dias após o início do tratamento			60 dias após o início do tratamento		
	Controle	Grupo TB+	Grupo TB−	Grupo TB+	Grupo TB+ no início do estudo	Grupo TB−	Grupo TB+	Grupo TB+ no início do estudo
	(n = 35)	(n = 35)	(n = 15)	(n = 14)	(n = 35)	(n = 23)	(n = 11)	(n = 35)
BMI, kg/m^2^	25,27 ± 3,59	18,21 ± 2,53*	19,60 ± 2,18*	19,40 ± 2,46*	19,49 ± 2,86*	20,64 ± 3,25*	20,41 ± 3,10*	20,53 ± 3,11*
DCT, mm	12,71 ± 4,99	5,11 ± 2,51*	6,28 ± 2,48*	5,87 ± 1,82*	6,13 ± 2,57*	7,42 ± 4,32*	7,15 ± 2,83*	7,35 ± 3,80*
AMB, cm^2^	55,15 ± 12,11	26,10 ± 7,92*	28,54 ± 9,86*	29,07 ± 9,24*	28,74 ± 9,69*	32,29 ± 12,37*	30,52 ± 12,09*	31,77 ± 11,94*
Albumina, g/dL	4,86 ± 0,19	3,64 ± 0,62*	3,99 ± 0,38*	4,02 ± 0,60*	4,02 ± 0,47*	4,27 ± 0,50*†	3,95 ± 0,37*	4,16 ± 0,48*†
PCR, mg/dL	0,16 ± 0,16	6,35 ± 4,12*	2,31 ± 1,88*†	4,33 ± 3,36*	3,66 ± 3,55*	1,95 ± 1,70*†	4,43 ± 3,69*‡	2,68 ± 2,71*†
PCR/albumina	0,03 ± 0,03	1,93 ± 1,58*	0,60 ± 0,52*†	1,22 ± 1,20*	0,99 ± 1,11*†	0,48 ± 0,44*†	1,20 ± 1,06*‡	0,70 ± 0,76
Se, μg/L	100,12 ± 12,11	80,13 ± 46,92*	93,55 ± 56,40*	77,31 ± 40,64*	88,26 ± 54,56*	104,53 ± 55,35	70,89 ± 38,66*‡	97,60 ± 54,59††

TB+ : cultura de escarro positiva ou baciloscopia de escarro positiva naquele momento do estudo

TB− : cultura de escarro negativa e baciloscopia de escarro negativa naquele momento do estudo

IMC : índice de massa corporal

DCT : dobra cutânea tricipital

AMB : área muscular do braço

PCR : proteína C reativa

Se : selênio

a Valores expressos em média ± dp

* p < 0,05 vs. controle

† p < 0,05 vs. grupo TB+ no início do estudo.

‡ p < 0,05 grupo TB+ vs. grupo TB−

†† p < 0,05 grupo TB+ vs. grupo TB+ no início do estudo

Teste de Tukey (variâncias iguais), teste de Games-Howell (variâncias diferentes).

A Tabela 4 apresenta a distribuição dos pacientes nos grupos TB+ e TB− em relação aos resultados dos testes bioquímicos e aos níveis séricos de selênio nos três momentos de estudo para determinar se havia associações. Para avaliar a associação entre os resultados dos testes bacteriológicos (cultura e baciloscopia) e os níveis séricos de selênio, foi usado o ponto de corte baseado na mediana dos resultados obtidos no grupo controle saudável.

**Tabela 4:** Distribuição dos pacientes nos grupos TB+ e TB− em relação aos resultados dos testes bioquímicos e aos níveis séricos de selênio nos três momentos do estudo.a

Variável	Início do estudo	30 dias após o início do tratamento			60 dias após o início do tratamento		
	Grupo TB+	Grupo TB−	Grupo TB+	p*	Grupo TB−	Grupo TB+	p*
	(n = 35)	(n = 15)	(n = 14)		(n = 23)	(n = 11)	
Albumina, g/dL				0,792			0,338
< 3,4	11 (100)	1 (33,3)	2 (66,7)		2 (100)	0 (0,0)	
3,4-4,8b	24 (100)	13 (54,2)	11 (45,8)		19 (63,3)	11 (36,7)	
>4,8	0 (0,0)	1 (50,0)	1 (50,0)		2 (100)	0 (0,0)	
PCR, mg/dL				0,617			0,683
< 0,3b	1 (100)	2 (100)	0 (0,0)		2 (100)	0 (0,0)	
≥ 0,3	34 (100)	13 (50,0)	13 (50,0)		21 (65,6)	11 (34,4)	
Relação PCR/albuminac				0,206			0,041
< 0,4	2 (100)	3 (30,0)	7 (70,0)		12 (75,0)	4 (25,0)	
0,4-1,1	11 (100)	5 (50,0)	5 (50,0)		9 (81,8)	2 (18,2)	
1,2-2,0	8 (100)	2 (50,0)	2 (50,0)		2 (50,0)	2 (50,0)	
> 2,0	13 (100)	3 (100)	0 (0,0)		0 (0,0)	3 (100)	
Selênio							
< ponto de corte^d^	24 (100)	9 (47,4)	10 (52,6)	0,518	12 (54,5)	10 (45,5)	0,027
≥ ponto de corte^d^	11 (100)	6 (60,0)	4 (40,0)		11 (91,7)	1 (8,3)	

TB− : cultura de escarro negativa e baciloscopia de escarro negativa naquele momento do estudo

TB+ : cultura de escarro positiva ou baciloscopia de escarro positiva naquele momento do estudo

a Valores expressos em n (%)

b Valores normais

c Usada para determinar o nível de riscos de complicações.^(^ ^13^ ^)^

d Baseado na mediana dos resultados obtidos no grupo de controles saudáveis

* Teste do qui-quadrado

## Discussão

No presente estudo, as características clínicas dos pacientes são semelhantes às descritas em outros estudos realizados em hospitais de referência para o tratamento da tuberculose em países em desenvolvimento, com altas taxas de alcoolismo e uso de tabaco.^(^ ^15^ ^)^

A relação entre tuberculose e desnutrição vem sendo revisitada, já que a desnutrição pode predispor à tuberculose ativa, e a tuberculose pode contribuir para a desnutrição.^(^ ^16^ ^)^ A média de perda de peso no grupo de estudo antes do início do tratamento da tuberculose foi de 11,03 ± 9,69 kg. Esse achado pode ser considerado ainda mais significativo quando categorizado pela porcentagem de perda de peso corporal, pois 63% dos pacientes apresentaram perda de peso ≥ 10%, que é considerada um fator predisponente para tuberculose.^(^ ^17^ ^)^

No presente estudo, a avaliação do estado nutricional com base em parâmetros antropométricos (IMC, DCT e AMB) confirmou o estado nutricional empobrecido do grupo de estudo, conforme descrito na literatura.^(^ ^18^ ^)^ Em qualquer infecção, existe uma interação complexa entre a resposta do hospedeiro e a virulência do microrganismo, que modula a resposta metabólica, bem como o grau e padrão de perda tecidual. Em pacientes com tuberculose, a redução do apetite, a má absorção de macronutrientes e micronutrientes e o metabolismo alterado levam a caquexia.^(^ ^16^ ^)^ No entanto, não se observou nenhuma associação entre os parâmetros nutricionais estudados e a conversão da cultura aos 60 dias de tratamento da tuberculose. Não obstante, constatamos que IMC, DCT e AMB baixos persistiram nos pacientes com tuberculose (mesmo naqueles cujos resultados se converteram em resultados negativos) após 60 dias de tratamento. 

Já se relatou o uso do IMC como indicador de nutrição na relação entre o estado nutricional e a tuberculose.^(^ ^19^ ^)^ A avaliação da DCT e da AMB em pacientes com tuberculose, no entanto, é menos frequentemente descrita na literatura. Não obstante, um grupo de autores^(^ ^20^ ^)^ descreveu diferenças no ganho de massa magra e massa gorda em pacientes com tuberculose após 6 meses de tratamento. Esse fato indica a importância não só de avaliar o ganho de peso total, mas também de diferenciar ganho de massa magra de ganho de massa gorda. 

No tocante aos testes bioquímicos estudados, constatamos que os níveis de albumina melhoraram durante o tratamento da tuberculose. Relatou-se que pacientes com tuberculose recém-diagnosticada apresentam níveis de albumina menores que os observados em grupos de controles saudáveis,^(^ ^18^ ^)^ o que corrobora os resultados do presente estudo. Em um estudo realizado na Tanzânia, os níveis de albumina de pacientes com tuberculose também aumentaram significativamente após 60 dias de tratamento da tuberculose, igualando-se aos níveis encontrados no grupo controle, o que vai de encontro a nossos achados.^(^ ^21^ ^)^ Em outro estudo realizado no Brasil, pacientes com tuberculose foram acompanhados durante 6 meses, e não se observou nenhuma melhora nos níveis de albumina ao longo do estudo.^(22)^

Níveis mais elevados de albumina foram considerados preditores de um desfecho melhor em pacientes com tuberculose pulmonar. A albumina também foi identificada como sendo um indicador do estado proteico quando se diagnostica a tuberculose.^(^ ^23^ ^)^ No entanto, as citocinas presentes durante a resposta de fase aguda (RFA) à infecção podem suprimir a síntese de albumina, reduzindo assim seus níveis circulantes. Portanto, é difícil interpretar níveis baixos de albumina em pacientes com tuberculose ativa sem outros parâmetros para avaliar a RFA e a desnutrição, já que níveis baixos de albumina podem refletir tanto RFA a infecção como deficiência proteica. Assim, é possível que a discrepância entre os resultados de estudos ocorra em virtude de variações do estado nutricional, da intensidade da RFA nas populações estudadas ou do pequeno número de pacientes incluídos. 

Como a síntese de PCR aumenta na resposta sistêmica do hospedeiro à infecção, foram observadas diferenças estatisticamente significantes entre os grupos TB+ e TB− no início do estudo, aos 30 dias de tratamento e aos 60 dias de tratamento, o que confirma a associação entre conversão bacteriológica e diminuição dos níveis de PCR. 

Um grupo de autores avaliou os níveis de PCR em pacientes com tuberculose pulmonar durante 6 meses de tratamento; 3 e 6 meses após o início do tratamento, houve redução significativa dos níveis de PCR.^(^ ^22^ ^)^ A PCR foi identificada como sendo um indicador importante para o diagnóstico de indivíduos com suspeita de tuberculose e baciloscopia positiva.^(^ ^24^ ^)^ Em nosso estudo, verificou-se uma associação estatisticamente significante entre relação PCR/albumina mais baixa e cultura negativa para micobactérias. Relatou-se que a relação PCR/albumina é maior em pacientes com outras doenças relacionadas com a RFA.^(^ ^14^ ^)^

A infecção tuberculosa é uma doença conhecida por induzir estresse oxidativo no organismo infectado, como a produção de espécies reativas de oxigênio (ERO) derivadas de radicais livres. Essas ERO estão relacionadas com disfunção na tuberculose pulmonar. Podem-se suprimir essas ERO por meio de enzimas antioxidantes, que removem radicais livres e protegem as células de dano oxidativo. Várias dessas enzimas, tais como a glutationa peroxidase, têm o selênio como elemento essencial.^(^ ^25^ ^)^ Assim, a redução da ingestão de micronutrientes (tais como vitaminas, zinco e selênio) leva a respostas imunológicas deficientes. 

Estudos mostram que pacientes com tuberculose ativa têm menores concentrações de vários micronutrientes, inclusive selênio, no sangue.^(^ ^26^ ^)^ No presente estudo, os indivíduos saudáveis apresentaram níveis de selênio mais elevados que os do grupo de estudo no início do estudo. Nos pacientes com tuberculose pulmonar, notamos uma associação entre cultura positiva e baixos níveis de selênio mesmo após 60 dias de tratamento. A deficiência de micronutrientes é uma causa frequente de imunodeficiência secundária e morbidade em virtude de infecções relacionadas, inclusive a tuberculose. Esse elemento traço tem um papel importante na manutenção de processos imunológicos e, portanto, pode ter um papel fundamental na defesa contra as micobactérias. Baixos níveis de selênio foram considerados um significativo fator de risco de micobacteriose em pacientes HIV positivos.^(^ ^27^ ^)^ Em um estudo com 22 pacientes com tuberculose pulmonar recentemente diagnosticados com escarro positivo,^(^ ^28^ ^)^ os autores observaram uma diferença significativa entre o grupo controle e o grupo de estudo no tocante aos níveis basais de selênio, como no presente estudo. No entanto, naquele estudo, não foram realizados testes bacteriológicos 60 dias depois. No presente estudo, é digno de nota que os níveis de selênio tenham permanecido baixos nos indivíduos do grupo TB+. Em um estudo realizado na Índia, um grupo de autores avaliou as concentrações circulantes de enzimas antioxidantes nas quais o selênio é um componente essencial e que são marcadores de estresse oxidativo em pacientes com tuberculose pulmonar.^(^ ^29^ ^)^ Os resultados revelaram menor potencial antioxidante por intermédio de baixos níveis de superóxido dismutase, catalase e glutationa, bem como aumento da peroxidação lipídica (malonaldeído) nos pacientes com tuberculose. No entanto, o potencial antioxidante e os níveis de selenoenzimas aumentaram com o tratamento, como se observou no presente estudo. 

Em outro estudo, realizado em Malawi,^(^ ^30^ ^)^ com 500 pacientes que haviam recentemente recebido diagnóstico de tuberculose pulmonar (incluindo 370 pacientes coinfectados por HIV), observou-se que deficiências de micronutrientes eram comuns em todos os pacientes, e 88% da amostra apresentava deficiência de selênio. Essas reduzidas concentrações de selênio também foram associadas à gravidade da anemia, que é comum em pacientes com tuberculose ativa. Sugere-se, portanto, que a deficiência de selênio pode contribuir para a anemia por meio do aumento do estresse oxidativo em pacientes com tuberculose. De acordo com um grupo de autores,^(^ ^5^ ^)^ uma intervenção de dois meses com vitamina E e suplemento de selênio reduziu o estresse oxidativo e aumentou a capacidade antioxidante total em pacientes com tuberculose pulmonar tratada. No entanto, naquele estudo,^(^ ^5^ ^)^ não houve associação entre suplemento de selênio e baciloscopia ou cultura negativa após 2 meses de tratamento. 

Em suma, em nosso estudo, observamos um estado nutricional ruim (com base no IMC, na DCT e na AMB) em pacientes com tuberculose pulmonar, mas esses parâmetros não se relacionaram com a conversão da cultura de escarro aos 60 dias de tratamento da tuberculose. A relação entre os níveis de PCR e albumina pode ser uma ferramenta útil para avaliar a conversão bacteriológica em pacientes com tuberculose. Além disso, os baixos níveis séricos de selênio após 60 dias de tratamento relacionaram-se com cultura e baciloscopia de escarro positivas. Nossos resultados corroboram os de outros estudos que mostraram melhora no estado imunológico de pacientes com tuberculose que receberam suplemento de selênio.^(^ ^27^ ^,^ ^30^ ^)^ Assim, apesar das limitações do presente estudo (amostra pequena de pacientes com tuberculose e inclusão de pacientes do sexo masculino apenas), nossos resultados sugerem que os níveis de selênio e a relação PCR/albumina podem ser usados como biomarcadores de resposta terapêutica na tuberculose pulmonar. Mais estudos são necessários para confirmar ou refutar nossos resultados. Além disso, estudos sobre a interação entre *M. tuberculosis* e níveis séricos de selênio são necessários para nos ajudar a entender se (e como) a tuberculose modula os níveis de selênio.
